# Caregivers’ perceptions, challenges and service needs related to tackling childhood overweight and obesity: a qualitative study in three districts of Shanghai, China

**DOI:** 10.1186/s12889-021-10744-6

**Published:** 2021-04-21

**Authors:** Yanting Wu, Xiaoying Ma, William D. Fraser, Mu Li, Wei Wang, Hefeng Huang, Myriam Landry, Yanhui Hao, Han Liu, Sonia Semenic, Yan Zhang, Haiqin Wang, Jingya Zhang, Jiale Yu, Xuena La, Congcong Zhang, Isabelle Marc, Hong Jiang

**Affiliations:** 1 International Peace Maternity and Child Health Hospital, School of Medicine, Shanghai Jiao Tong University, 910 Hengshan Road., Shanghai, 200030 China; 2 Obstetrics and Gynecology Hospital, Institute of Reproduction and Development, Fudan University, Shanghai, 200011 China; 3 School of Public Health, Key Laboratory of Health Technology Assessment, National Health Commission of the People’s Republic of China, Fudan University, Mailbox 175, No. 138 Yixueyuan Road, Shanghai, 200032 China; 4 Shanghai Center for Disease Control and Prevention, No.1380 West Zhongshan Road, Shanghai, 200336 China; 5 Department of Obstetrics and Gynecology, Université de Sherbrooke Centre de recherche du CHUS, CIUSSS de l’Estrie – CHUS, 3001, 12e avenue Nord, Sherbrooke, QC J1H 5N4 Canada; 6 School of Public Health, China Studies Centre, University of Sydney, Room 313, Edward Ford Building, Sydney, 2006 Australia; 7 Department of Pediatrics, CHU de Quebec Université Laval research center, 2705 Boulevard Laurier, G1V 4G2 Quebec City, Quebec Canada; 8 Ingram School of Nursing, McGill University, 680 Sherbrooke West, Suite 1800, Montreal, QC H3A 2M7 Canada; 9 Jiading District Center for Disease Control and Prevention, No.264 Tacheng road, Shanghai, 201800 China; 10 Shanghai Health Promotion Center, No.122 South Shaanxi road, Shanghai, 200040 China

**Keywords:** Childhood overweight and obesity, Caregiver, Beliefs, Challenges, Service needs

## Abstract

**Background:**

Childhood overweight and obesity (OWO) has become a major public concern worldwide including in Shanghai, one of the most developed areas of China. Understanding perceptions and challenges of tackling childhood OWO among caregivers of children is critical to provide services in need.

**Methods:**

A qualitative descriptive study including in-depth interviews with seven parents and six focus group discussions with a total of 32 parents or grandparents of children zero to 6 years of age. Participants lived in three districts of Shanghai and indexed children included both those with OWO or non-OWO children. Data were analyzed using qualitative thematic analysis.

**Results:**

Caregivers tended to underestimate children’s weight status, and to regard chubby children as a sign of good parental care. Some caregivers even suggested that there were positive effects of childhood overweight. Caregivers identified a number of challenges to prevention of OWO in children, including difficulties in controlling dietary intake or increasing children’s physical activities; discordant views between parents and grandparents, and barriers to accessing professional guidance. Caregivers desired more detailed advice regarding children’s nutrition intake and physical activity, and preferred online approaches.

**Conclusions:**

Misconceptions regarding childhood overweight were found in caregivers of children in Shanghai. Professional guidance on childhood weight control for caregivers is desired via digital applications such as mobile phone applications and social media.

## Background

Childhood overweight and obesity (OWO) has become a major public health concern worldwide. Forty-one million children under the age of five were overweight or obese in 2016 and nearly half of them lived in Asia [1]. As other countries, in less than one generation, China has experienced a substantial rise of childhood OWO [2]. The prevalence of obesity among children under 7 years of age between 1985 and 2005 in China increased from 0.9 to 3.2% [3]. A national survey conducted in 2016 in nine cities (including Shanghai) reported the overall prevalence of overweight and obesity of children aged from 0 to 7 years to be 8.4 and 4.2%, respectively. The data for Shanghai found the prevalence of overweight and obesity to be higher than the national rate, at 9.1 and 4.4%, respectively [4].

Childhood OWO has short and long-term adverse impacts on children’s health. These included increased risks for delayed neurodevelopment as well as the development of chronic diseases such as diabetes, heart disease and cancers [5]. Many findings have highlighted the importance of preventive measures early in the life cycle to prevent childhood OWO [6]. Caregivers hold an important role as gatekeepers in promoting healthy eating and active living to prevent OWO [7]. In some cultures, such as in China, grandparents often play a primary role in early childcare [8]. In a family context, food represents nurturance thereby serving an important role in bonding between Chinese caregivers and children, which may be disrupted when putting children on a diet [9]. Therefore, effective interventions to tackle childhood OWO must involve all family members who provide primary care, including parents or grandparents. In order to provide effective services for childhood OWO prevention, it would be important to understand in more depth the perceptions of primary caregivers regarding childhood OWO and the challenges that they face in achieving healthy weight gain of children.

The aim of this study was to gain in-depth and multiple understandings of perceptions of childhood OWO and challenges related to childhood OWO prevention and management among caregivers in three districts of Shanghai, China. The findings of this study served to inform the design of a multicenter intervention study that is currently underway in Shanghai.

## Methods

### Study design

A qualitative descriptive study design [10] was used to explore caregiver perceptions of childhood OWO in depth. Data were collected via a combination of focus group discussions (FGDs) and in-depth individual interviews (IDIs), to enhance data richness [11]. Findings reported in this paper were part of a larger qualitative study to inform the development of a multi-faceted intervention program to prevent childhood OWO. Semi-structured interview guides were used to inquire about caregivers’ views about childhood OWO, as well as challenges, facilitators and informational needs related to children’s diet and exercise.

### Participants and recruitment

Participants were purposely selected from Xuhui, Changning and Fengxian Districts, representing high, medium, and relatively lower family income areas of Shanghai, China. In Xuhui and Changning districts, participants were recruited from the child health outpatient clinics of the tertiary maternal and child health care hospital in each district. In Fengxian district, participants were recruited from one community health care center that serviced a large population in the surrounding area, as there was no child health clinic in the district’s maternal and child hospital. Eligible participants were primary caregivers, including parents and grandparents, of children from birth to 6 years old with normal body weight or OWO. Inclusion criteria included the ability to communicate in Mandarin, and acceptance to participate in the study. Caregivers of children attending child health care services in the study sites were approached and invited by trained health care providers to join the study. Potential participants were first queried regarding their willingness to participate in the study, and if so, were asked about their availability to participate in an in-depth individual interview (IDI) or attend a focus group discussion (FGD). Those who were open to ‘either’ option but were not available at the time of scheduled FGDs, were recruited to participate in the IDIs.

Body mass index (BMI) is widely used to screen for OWO children over 2 years of age as well as adults [12, 13]. The weight and length/height of children were drawn from their routine child health care records, measured by doctors using a standardized procedure for the routine child health visit of Shanghai. For children over 2 years old, a BMI above the 85th percentile is defined as overweight and 95th percentile as obesity based on the growth chart developed for Chinese children and adolescents [14, 15]. A weight-for-length value above the 95th percentile is defined as overweight for children under 2 years old [16]. Caregivers’ weight status was self-reported before the interview or FGD was initiated. For caregivers, a BMI value above 24 kg/m^2^ was defined as OWO [17].

### Data collection

Data collection took place between October and December 2017. All participants were informed of the purpose of the study and written consent was signed before the IDIs and FGDs took place. Semi-structured interview guides for the FDGs and IDIs were developed by the research group, which included qualitative research experts and maternal and child health care researchers. The study’s interview guides were piloted with two parent volunteers and modified based on their feedback. Each IDI or FGD was conducted in a quiet and separate room by two researchers with experience in child health care and qualitative research methodologies. One researcher facilitated the interview using the semi-structured interview guide. The other researcher took notes on the main reflections from the interviews/discussions as well as the participants’ non-verbal communication (e.g., body movements, facial expressions) during the interviews [18]. The IDIs and FGDs were digitally-recorded, and lasted from 45 to 90 min.

The groups for the FGDs were composed of a mixture of caregivers from the medium income (Xuhui) and high income (Changning) Districts. FGDs were held with parents and grandparents separately to promote group homogeneity and generate more focused discussions around key issues. Six FGDs were carried out: two with parents of OWO children, two with parents of non-OWO children, and two with grandparents of either normal weight or OWO children. For each of these combinations, one FGD was held with caregivers from Fengxian District, and the other was held jointly with caregivers from Xuhui and Changning Districts. Each FGD was comprised of five to six participants, totaling 32 individuals. In addition, IDIs were conducted with seven caregivers (six mothers and one father), three of whom had OWO children. Sample size guidelines for qualitative thematic analysis suggest a minimum of four to five dense interviews for a research question, to be able to identify recurrent patterns and themes in the data [19]. As no new findings emerged following analysis of the FGDS and IDIs, no additional interviewing was required. Therefore, a total of 39 participated in the study.

### Data analysis

All digital recordings were transcribed verbatim in Mandarin and saved as Microsoft Word documents. Data were analysed using a combined deductive and inductive thematic analysis approach, following the steps of 1) familiarization with the data, 2) coding, 3) generating initial themes, 4) reviewing themes and 5) defining and naming the final themes [20]. Data were analyzed using Nvivo10. The main interview guide questions initially served as broad overarching themes. First, two researchers read and re-read the FGD and IDI transcripts to become familiar with the data. The researchers then independently coded the data by highlighting words or phrases that reflected thoughts and issues corresponding to the main interview questions. The codes from the FGD and IDIs were combined into one data set for analysis, as the same interview questions were asked for both. The initial codes were then examined to identify patterns of meaning, labeled and merged into initial themes and subthemes. The two researchers compared and discussed differences in their themes and coded data until agreement was reached. Next, the preliminary themes were checked back against the original dataset to validate their goodness of fit. The final themes and subthemes were defined and named in group discussion with the research team. Themes and representative quotes from the raw data were translated into English by the researchers who were native Mandarin Chinese speakers with fluency in English and experience in translation. Table 1 presents the final themes, subthemes and codes from the data analysis.

**Table 1:** Themes, subthemes and codes emerged from the data

Main themes	Subthemes	Codes
Caregiver perceptions of children’s weight	Underestimation of children’s weight status	Thin, chubby, little fatties
	Chubby children as a sign of good parental care Normal for children to be chubby	OWO as sign of good parenting and feeding competence; chubby children a sign of good health Chubby children are normal; will become thinner when growing up.
Caregiver beliefs about the health consequence of childhood OWO	Negative effect of OWO on short term health Negative effects on long-term health	Bad for health; slower development, lacking confidence; Diabetes, hypertension, metabolic diseases
	Positive effects of childhood overweight	Less likely to get sick
Challenges related to dealing with childhood OWO	Difficulties controlling child’s dietary intake	Using food to stop children from crying; grandparents’ indulgent feeding practices; picky eaters; temptation of junk food.
	Difficulties in increasing children’s physical activities	Lack of awareness among grandparents; lack of venues and facilities for physical activities
	Inadequate guidance from health professionals	Inadequate communications and information from health providers
Information needs regarding childhood OWO prevention	Demand for more detailed advice on childhood feeding and physical activity	What foods to add at different month of age; what kind of and what level of exercise is suitable for children of different age
	Access to reliable information	Public hospitals; information from professionals
	Preference of online information	Not easy to get people together to attend lectures; Online information, e.g. via WeChat, is convenient.

## Results

General characteristics of participants are presented in Table 2. The average ages of parents and grandparents were 32 and 61 years old respectively. The majority of the participants were mothers and grandmothers. Most of participants held Shanghai permanent residence status. The highest education level of most parents was bachelor or above. Eight of 29 parents and half of the grandparents were overweight or obese. The median age of children was 2 years (range from 0.2 to 6.0 years), with OWO children being younger on average (median 1.5 years, range from 0.2 to 6.0) years than non-OWO children (median 2.6, range from 0.3 to 6.0 years), respectively.

**Table 2:** General characteristics of Participants

Group	IDI		FGDs		
	Parents (n)		Parents (n)		Grandparents (n)
	Child of OWO	Child of Non-OWO	Child of OWO^a^	Child of Non-OWO^a^	Combined Children of OWO and non-OWO
**Total**	3	4	11	11	10
**Age (Mean ± SD, years)**	31.0 ± 2.7	32.3 ± 4.4	32.4 ± 3.2	32.5 ± 4.3	61.0 ± 4.7
**Gender**					
Female	3	3	8	8	8
Male	0	1	3	3	2
**Registered permanent residence**					
Shanghai	2	2	9	9	8
Other provinces	1	2	2	2	2
**Highest Education**					
Less than bachelor degree	0	0	0	0	8
Bachelor degree and above	3	4	11	11	2
**BMI**^**b**^					
Underweight	0	1	0	0	0
Normal	1	2	7	10	5
OWO	2	1	4	1	5

^a^*OWO* Overweight and obesity

^b^BMI classification follows the Determination of Adult Weight as released by National Health and Family Planning Commission, Underweight (<18.5), Normal (18.5 ~ 23.9), Overweight (24.0 ~ 27.9), Obese (28.0~). Children’s BMI was calculated based on the weight and height/length measurements by health professionals during the routine child health care check-up. Parents and grandparents’ BMI was calculated based on the self-reported weight and height

### Themes

Findings were grouped under four main themes: (1) caregiver perceptions of children’s weight, (2) beliefs about the health consequences of childhood OWO, (3) challenges related to tackling childhood OWO, and (4) information needs regarding childhood OWO prevention. We did not identify differences between districts (Xuhui, Changning and Fengxian) in caregivers’ views, challenges and informational needs related to OWO prevention and control.

#### Views of caregivers about childhood overweight and obesity

Caregivers frequently misperceived children’s weight and normalized infant chubbiness. Some felt that chubby infants reflected good caregiving.

##### Underestimation of children’s weight status

Many caregivers, especially the grandparents of overweight children, underestimated their children’s weight status. Some of them perceived their children who were of normal weight as ‘underweight’ while others perceived their overweight children as ‘normal weight’:

*“My mother-in-law is persistent, and she feels that my baby is ‘not fat’, even if he is overweight.*

*“My parents say the same.” (FDG-FX-parents of OWO children)*

*“Well, a fat child is just a few pounds heavier than the one with normal weight, looks chubby.” (IDI-FX- a parent of children of normal weight-02)*

Caregivers commonly used words with a negative connotation such as “thin” to describe their children who were of normal weight:

*“His weight is normal, and he is not heavy. As whole, he looks thin.” (IDI-CN-a parent with a child of normal weight -02).*

Conversely, they were likely to use words with a positive connotation, such as “chubby”, or “little fatties” to describe their overweight children:

*“(I am) very proud of our child being overweight. We call them [OWO children] ‘little fatties’”. (FDG-XH-parents of OWO child)*

##### Chubby children as a sign of good parental care

Some caregivers of OWO children viewed overweight as a sign of good health and parental care and feeding competence:

*“My parents do not think it is necessary for children to control weight. And they think having an overweight baby is a sign of good parental competence.” (FDG-FX-parents of OWO children).*

##### It’s normal for children to be chubby

Additionally, parents of both OWO or normal-weight children perceived that children would turn to a normal weight as they grew taller and older:

*“I think it is normal and okay that the baby is chubby. He will become thinner after he becomes taller. It doesn't matter that he is chubby now.” (IDI-XH-a parent of children of normal weight)*

*“Many children will have normal weight after they wean or they become older.” (IDI-FX-parents of OWO children-01)*

#### Caregivers’ beliefs about the health consequence of childhood OWO

Most caregivers believed that childhood OWO could have negative effects on children’s short term as well as long-term health and should be a concern. However some thought childhood OWO was not a problem as long as children looked healthy, or that it may even benefit children’s health.

##### Negative effect of OWO on children’s short-term health

In the short term, caregivers reported that being overweight or obese would lead to slower physical development:

*“I have a friend. His son is very fat. It seems not convenient for the baby to turn over. He struggled to turn over.” (FDG-XH-parents of children with normal weight)*

*“I think [being obese] will have an effect on the development [of the children]. If the baby is fat, he/she will lag behind in the development of walking.”*

*“Firstly, from the perspective of children's growth and development, being overweight or obese places a heavy burden on the bones.”*

Caregivers also perceived that being overweight or obese could impact children’s cognitive or psychological development, and jeopardize their self-confidence:

*“My daughter is fat. Compared with other kids in the class, she seems to be slow in psychological development, not to mention her clumsiness in physical activities." (FDG-FX-parents of OWO children)*

*“I think being obese will affect children’s intelligence … ”*

*“Obesity may have some impact on his later psychological wellbeing, because obesity may make the child feel lacking in self-confidence.” (FDG-XH- parents of OWO children)*

##### Negative effect of OWO on long-term health

Most caregivers thought that childhood obesity would lead to the development of chronic illnesses such as diabetes, hypertension and metabolic diseases in childhood, youths and adulthood:

*“It seems OWO children would get diabetes or hypertension when they are still young.”*

*“I think it's not good for a kid to become very heavy, as he/she will face a higher risk of diabetes or other diseases in adulthood.” (IDI-XH-parents of children of normal weight-01)*

##### Positive health effects of childhood overweight

Conversely, some parents and grandparents viewed overweight as a sign of good health. They believed that chubby babies were less likely to get sick and their immunity would perform relatively better.

*“[Being chubby] has its merits. Before going to the kindergarten, although my daughter was chubby, her constitution was not bad … Still, fatness endows her with good immunity, and she seldom fell sick."(FDG-FX-a parent of an overweight child)*

#### Challenges related to tackling childhood overweight and obesity

Some parents reported that they had tried to control their children’s weight. However, they met with difficulties which prevented them from changing behaviors, including challenges managing children’s dietary control and physical activity, and a lack of professional guidance.

##### Difficulties controlling children’s dietary intake

Caregivers understood that a healthy diet, including a balanced dietary intake and appropriate food portions, was important for maintaining a healthy weight, but they reported many barriers in achieving their dietary goals. One important issue was the use of food to stop children crying. Some parents disclosed that their children had a good appetite and demanded food all the time. They felt that they lacked the tactics to deal with the situation and needed professional guidance:

*“Once he cried, his grandmother would hold him … She [grandmother] was very tired... so in order for her not having to hold the baby I would give him food [to stop him crying].”*

One mother mentioned that her obese baby of 5 months old had an abdominal hernia which would get worse when crying. However, the baby would not settle unless she got more food. The mother had to feed more milk until the baby became satisfied, even though it exceeded the recommended amount by the doctor and reached 300 ml per meal.

*“The doctor advised me to reduce the amount of milk for my baby but I couldn’t follow his advice. Because my baby will keep crying if she feels hungry, which is dangerous because she has hernia.”*

*(FDG-FX-parents of OWO children)*

Some caregivers commented that their children were “picky” eaters and didn’t like healthy foods, and found it hard to change children’s food preferences.

*“[My grandchild] does not like vegetables or fruits.”*

*“My grandchild likes to eat meat only. Sometimes I fed him with vegetables when he’s playing, but he would spit it out.”*

*“I am anxious every day because my grandchild does not want to eat vegetables ... or eggs.”*

*(FDG-FX-grandparents)*

At the same time, caregivers found that junk food was often more appealing to children, while healthy food was less attractive to children. Therefore, it was difficult to have children stick to healthy foods.

*“Fried food. They like it very much. Such as potato chips … ”*

*“Nowadays, there are many kinds of foods arousing children's curiosity and grandparents have no excuse not to give to them when their grandson/granddaughter wants to try...”*

*(FDG-FX-grandparents)*

Parents described the inability to control the food intake of their children offered by indulgent grandparents. They mentioned that their children were fed with unhealthy food and unrestricted portions from grandparents. This made it hard for parents to cultivate appropriate dietary habits for their children.

*“The old people feed the children, and keep them eating, and there is a kind of hunger called ‘grandma thinks you are hungry’.”*

*“The grandparents would think about the children in their own way. For example, when the old people feel cold, they think the children also feel cold. Thus they would let the children put on more clothes.”*

*(FDG-FX-a parent of a child of normal weight)*

*“The old people are very persistent and contributed half to the obesity … They cook the foods that the child likes.”*

*“The old people occasionally pay attention to information on the child's diet or activity, but in practice, they fail to follow it, and when they find that the child does not like eating healthy foods, they will not guide him to eat well and will finally give the child what he likes.”*

*(FDG-FX-parents of OWO children)*

##### Difficulties in increasing children’s physical activities

Some parents divulged that grandparents did not recognize the importance of physical activities in weight management and would not even allow their children do any housework appropriate for children, such as putting away their toys. For example, a few parents of obese children from Fengxian District mentioned that their children were over-protected by the grandparents and usually did nothing by themselves at home.

*“(grandparents get everything done?) In all respects, [grandparents] do not allow my boy to do any housework. We (parents) secretly let him do some housework.”*

*(FDG-FX-a parent of an OWO child)*

Parents also described barriers to children’s physical activities outside of the home. They mentioned that due to the lack of venues and facilities for physical activities in their communities, they were only able to take their child for walk, but this would depend on the weather conditions.

*"It is cold these days, so we did not take them out. In summer, we would take them out for a walk almost every day.”*

*“Sometimes we would go for a walk in the summer, yet it wouldn’t be possible in the winter because of the strong wind."*

*(FDG-FX-grandparents)*

##### Inadequate guidance from health professionals

Participants reported receiving inadequate information from medical or community health resources.

*“Doctors will tell us something about it, but just say it (the weight of my child) is relatively normal, heavier or lighter. It (the community center) is a place of health examination and they will not give detailed suggestions, just roughly, because it (overweight) can't be regarded as a disease.”*

*(IDI-CN-a parent of an OWO child-01)*

Some caregivers indicated that the health providers advised them to do passive exercises to control the weight of babies, but they were not able to give any detailed guidance on how to do it due to their large service volume. One parent reported that they were simply asked to search the video on the Internet.

*“At 4 months, [the doctor] said that [the baby] should do some passive exercise, ... but [the doctor] asked me to search online by myself ...”*

*“The doctor advised me to do more passive exercise ... but did not say [how to do].”*

*(FDG-FX-parents of OWO children)*

*"Sometimes doctors do not explain in details because of the large number of people waiting in child health clinics.”*

*(IDI-CN-a parent of a child in normal weight-01)*

#### Information needs regarding childhood OWO control and prevention

Caregivers face many difficulties to control their children’s weight and prevent OWO in their children. To overcome these barriers, caregivers mentioned that they needed more detailed information on child feeding and physical activities, and that informational source should be reliable with a preference for online approaches.

##### Need for more detailed information on feeding and physical activities

Many mothers mentioned that they would like to know more about feeding their children, such as when to add a specific complementary food and how to keep a balanced diet for children.

*“I want to know what I can feed him to keep a balanced diet.”*

*(IDI-CN-a parent of children of normal weight-01)*

*“I am not sure what food to add at different month of age. I have seen one [piece of information] before, which suggested that babies should not eat salt or honey before six months of age. I think I need pay more attention to such information.”*

*(IDI-FX-a parent of an OWO child-01)*

Some mothers also mentioned that they needed to know what kinds of activities were suitable for the development of children at different specific development period.

*“At least the information can contain detailed contents. For example, what kind of and what level of exercise is suitable for children of different ages? Whether it is excessive to do such exercise for children of certain age? How to do the exercise and how long should it be done? We need details or written things to refer.”*

*(IDI-CN-a parent of a child of normal weight-02)*

Many parents mentioned they preferred information and communication provided by health providers or authorized by hospitals and health institutions. All of them emphasized the importance of the reliability of information resource and service provision.

*“[I hope the source of the information] To be reliable, such as from public hospitals.”*

*(IDI-CN-a parent of a child in normal weight-01)*

*“[I will trust] The information from professional doctors or a professor.”*

*(IDI-XH-a parent of a child of normal weight-01)*

As for the delivery of reliable information, many caregivers preferred modern communication technology platforms to face-to-face consultation. They mentioned that WeChat (a Chinese social media platform similar to WhatsApp) was a popular communication platform, and it was convenient for them to read the tips published by the official accounts of the authorized health institutions through it.

*“It’s not easy to get people together to attend lectures, and the information on the Internet is more convenient … Now I often use WeChat, and will be definitely willing to read useful information … Online information is convenient for everyone.”*

*(IDI-XH-a parent of a child of normal weight-02)*

*“Either official accounts of hospitals or doctors’ WeChat IDs would be useful.”*

*(IDI-FX-a parent of a child of normal weight-01)*

## Discussion

Our findings revealed that many caregivers underestimated their children’s weight status. Some even viewed overweight as a sign of good parental care and feeding competence, and believed that their children would turn to a normal weight as they grew taller. Some parents found it difficult to control their children’s weight due to a lack of detailed professional guidance. Caregivers expressed the need for reliable sources of information to promote appropriate feeding and physical activity of children.

Our observation of the underestimation of child OWO by caregivers is similar to the findings of a systematic review, in which 32.3% (18,656/57,700) of children were classified as overweight or obese, but only 9.5% (5501/57,700) of children were perceived as overweight or obese by their mothers [21–24]. Previous studies have shown that the factors associated with caregivers’ misperception of children’s weight status included low maternal education, overweight mothers, male children, ethnicity and the social environment [21]. The culture preference of “fat” children among grandparents in China was reported in previous studies [8, 25]. Such beliefs largely stem from the Chinese historical context of famine and poverty, in which being slim represented poverty and poor health [8]. Therefore, some grandparents regarded overweight as a sign of good parental care and feeding competence. It might also be a sign of Chinese culture preferring “fat” children. For example, Amiri and Dapi et al. reported that overweight people were perceived as ‘strong’ in the Chinese culture [26, 27]. The caregivers also believed that children’s weight would be distributed more evenly and become normal as the child grew, a belief also reported in other studies [28, 29]. However, this ill-informed view of children’s weight problems might lead to a lost opportunity to adopt appropriate child care and feeding practices to prevent OWO [30]. These findings highlight the need to help caregivers establish appropriate understanding of childhood OWO.

Almost all caregivers recognized some short and long-term impacts of childhood OWO on health. However, parents faced many barriers when attempting to control their children’s weight within a normal range. This is consistent with previous studies [8, 26, 31–33], in which difficulties in not offering unhealthy foods and overfeeding their children were also predominant barriers reported, in addition to picky eating and the temptation of junk food. These findings suggest the necessity of providing relevant health promotion information on healthy child feeding during child health care consultations, and involving grandparents in the health promotion. Our study found that parents felt the need for additional professional guidance regarding child feeding practices, particularly when children had medical conditions. Our results also indicated the limited capacity in tackling weight control among health care providers. Training for health professionals and ensuring sufficient time to fully discuss these issues with the parents are needed in order to establish the confidence and skills in dealing with specific conditions of child weight control. Furthermore, referral services are necessary for families faced with complicated situations of child weight management.

As reported in other studies, parents in this study expressed needs for reliable information on how to improve the health of their children [33]. As digital communication technology develops, modern communication devices such as mobile phones and tablets have universal coverage in China [6, 34]. Mobile phone applications and social media platforms such as WeChat and public accounts of health authorities in Weibo are popular, and have potential to deliver health information to parents and grandparents. However, one study has found most of maternal and child health APPs in China were produced by commercial entities, focusing more on commercial activities than delivering credible and valid information [35]. Platforms and contents produced by health authorities with non-profit purpose, e.g. professional associations, public hospitals, are in a better position to meet the needs of knowledge dissemination.

This qualitative study contributes to filling in the gap in understanding regarding the perceptions and challenges of childhood overweight and obesity prevention and management among caregivers, which could not be obtained in quantitative studies. It has several limitations. Since Shanghai is one of the most developed areas of China, the findings might not be representative of other areas of China with different social economic status. Further, this research did not include perceptions, challenges and suggestions for childhood OWO prevention and control from the perspective of service providers.

## Conclusions

Misperceptions regarding childhood overweight and obesity were found among some caregivers of Shanghai children whose ages ranged from birth to 6 years. Detailed professional guidance for caregivers to prevent and control childhood OWO is needed. Reliable information delivered by health professionals through digital health applications such as APPs and social media has the potential to disseminate knowledge and build caregivers’ capacity for childhood weight control.

## Data Availability

Supporting data can be obtained from the corresponding author.

## Abbreviations

OWO: Childhood overweight and obesity;
FGDs: Focus group discussions;
IDI: In-depth individual interviews
